# Association of dual use of cigarettes with obstructive sleep apnea assessed by the STOP-Bang score

**DOI:** 10.18332/tid/169727

**Published:** 2023-09-13

**Authors:** Seung Hyun Lee, Seung Hoon Kim

**Affiliations:** 1 Department of Education and Training, Nowon Eulji Medical Center, Eulji University School of Medicine, Seoul, Republic of Korea; 2 Department of Preventive Medicine, Eulji University School of Medicine, Daejeon, Republic of Korea

**Keywords:** cigarette smoking, electronic cigarettes, dual use of cigarettes, obstructive sleep apnea, snoring

## Abstract

**INTRODUCTION:**

Although previous studies have addressed the association between smoking and obstructive sleep apnea (OSA), there are few studies on the association between electronic cigarette use and OSA. Thus, we aimed to evaluate the association between the dual use of electronic and conventional cigarettes and OSA.

**METHODS:**

Data from 7350 participants of the 2019–2021 Korean National Health and Nutrition Examination Survey were analyzed in this population-based study. The STOP-Bang score was calculated using eight items: snoring, tiredness, observed apnea, high blood pressure, body mass index, age, neck circumference, and sex. The main independent variable was smoking behavior. A multiple logistic regression analysis was performed. Subgroup analysis was conducted to analyze the association between smoking behavior in detail and OSA, and stratified analyses were additionally performed.

**RESULTS:**

Of the 7350 participants, 417 (5.7%) had a high risk of OSA, according to the STOP-Bang score. Compared to the non-smoker group, the dual user group had a 2.46-fold increase in the odds of OSA (adjusted odds ratio, AOR = 2.45; 95% CI: 1.04-5.79). Current non-smokers who were dual users in the past had increased odds of having OSA (AOR=3.61; 95% CI: 1.32–9.92). In the stratified analyses, dual cigarette use was significantly associated with OSA in females and those with a low physical activity level.

**CONCLUSIONS:**

Dual users and cigarette-only users had an increased probability of developing OSA. Even if they are not currently smoking or vaping, individuals who were dual users in the past were associated with a higher risk of OSA. The association between dual cigarette use and OSA was more pronounced in females and those with a low physical activity level. While intervening for obstructive sleep apnea or investigating risk factors, new smoking methods such as vaping and dual use should be considered along with conventional smoking.

## INTRODUCTION

Obstructive sleep apnea (OSA) is the most common sleep-related breathing disorder. The prevalence of OSA in China, Spain, Korea, Sweden, the United States, Japan, and India between 1993 and 2013 was 22% and 17% in males and females, respectively^1^. Over 90% of patients with OSA are misdiagnosed; therefore, most of them remain untreated^2^. OSA is a growing health problem that is associated with various complications such as daytime fatigue, neurocognitive effects, headaches on awakening, fetal growth retardation, and disruption of bed partners’ sleep quality. If left untreated, OSA can be accompanied by glaucoma, cardiovascular events, stroke, and pulmonary hypertension^3^. Therefore, to prevent health deterioration due to the complications of OSA, identifying the risk factors for OSA is an important public health challenge.

The gold standard for diagnosing OSA is by using an overnight polysomnogram; however, it is time-consuming, expensive, and requires a professional examiner. Therefore, several alternate, simple, and reliable methods were devised to identify individuals at high risk for OSA^4-6^. However, even these were complex or time-consuming and required upper airway evaluation. The STOP-Bang questionnaire, developed in 2008, is a simple and useful tool for screening OSA risk groups^7^. The STOP-Bang questionnaire is a self-reported questionnaire with four yes/no questions about snoring, tiredness, observed apnea, and high blood pressure, and four additional demographic questions concerning BMI (≥35 kg/m^2^), age (>50 years), neck circumference (>40 cm), and sex (male). Each question is assigned 1 point; thus, the total number of points ranges 0–8. Originally, the STOP-Bang scores were classified as: ≥3 for moderate-to-severe risk, and <3 for low risk of OSA. This tool has a sensitivity of ≥84%, 93%, and 100% and a specificity of 56.4%, 43%, and 37% for detecting any OSA, moderate to severe OSA, and severe OSA, respectively^8^. Owing to its high sensitivity and simplicity, STOP-Bang has been widely used in diverse populations^8,9^.

Male sex, older age, familial aggregation, obesity, central body fat distribution, large neck girth, upper airway abnormalities, congestive heart failure, type 2 diabetes mellitus, and stroke are well-known risk factors for sleep apnea^10^. Several recent studies have found an association between smoking and alcohol use and sleep apnea or snoring^11^. Current and former smoking, as well as heavy smoking, are associated with OSA^12^. The effects of smoking on OSA and their mechanisms have been studied; although it is still a hypothesis, the mechanisms may include alterations in sleep architecture, impairment of upper airway neuromuscular function, higher arousal index, and upper airway inflammation^14^. However, only a few studies have investigated the association between OSA and electronic cigarette (e-cigarette) use, even though e-cigarettes are becoming increasingly popular^14^. Since e-cigarettes are considered healthier alternatives to cigarettes, their use is increasing; hence, they are used to reduce or quit cigarette smoking^16^. Despite such beliefs, there is still a lack of information about smoking risks according to the type of cigarette, including electronic cigarettes. Thus, we investigated the association between smoking behavior including smoking status, types of cigarettes and OSA, as determined by the STOP-Bang score.

## METHODS

### Study population and data

This secondary dataset analysis was performed on the cross-sectional 2019–2021 Korean National Health and Nutrition Examination Survey (KNHANES) because the STOP-Bang questionnaire was administered only during this period. The KNHANES is a survey of national health conditions, nutritional intake, and dietary habits conducted by the Korea Disease Control and Prevention Agency (KCDA) in Korea. More information about the design and content of the KNHANES can be found on its webpage (knhanes. kdca. go. kr/knhanes/eng/index. do). The KNHANES protocols were approved by the Institutional Review Board of the KCDA (IRB No. 2018-01-03-C-A), and the research complied with the tenets of the Declaration of Helsinki for medical research involving human subjects. Informed consent was obtained from all the participants. Since the KNHANES is a survey conducted by the government for public welfare, ethics approval for the secondary dataset analysis of KNHANES is waived by the Bioethics and Safety Act of 2015.

A total of 22559 participants were included in the 2019–2021 KNHANES. As the survey on snoring, tiredness, and observed apnea was not conducted for respondents aged <40 years, only participants aged ≥40 years were included. Among them, 7350 participants (3226 male and 4124 female) were included in the present study after excluding participants with missing data.

### STOP-Bang score

The STOP-Bang score was used to screen for OSA. This questionnaire comprises eight items. Each item receives 1 point when conditions are satisfied, and the types of conditions are: 1) loud snoring, 2) daytime tiredness, 3) onlooker-observed cessation of breathing during sleep, 4) high blood pressure (BP) or consumption of medication for high BP, 5) body mass index (BMI) ≥35 kg/m^2^, 6) age >50 years, 7) neck circumference >40 cm, and 8) male sex. In this study, the STOP-Bang algorithm with a 2-step scoring strategy was used to identify the association between the high-risk groups for OSA and smoking behavior. In the first step, those with STOP-Bang scores ≥5 were classified as the high-risk group for OSA. Subsequently, among the intermediate group with STOP-Bang scores of 3–4, participants who were male, had a BMI >35 kg/m^2^, or neck circumferences >40 cm were also classified as the high-risk group (second step)^16^.

### Smoking behavior

The main independent variable in this study was smoking behavior which comprised current cigarette or e-cigarette smoking and a history of smoking. Individuals who used both e-cigarettes and cigarettes during their lifetime were classified as dual users, whereas those who used cigarettes only were classified as cigarette only smokers. E-cigarette users included users of e-cigarettes or liquid e-cigarettes containing nicotine. Because only a very small number of participants used e-cigarettes only during their lifetime (n=1) and this study was aimed at deriving the association between vaping and sleep disorders, we included the e-cigarette only users in the dual users. Finally, those who never used cigarettes or e-cigarettes during their lifetime were classified as never smokers. Based on these categories, the participants were divided into three groups: dual users of e-cigarettes and cigarettes, cigarette only users, and never smokers.

For the subgroup analysis, never smokers were classified according to exposure to secondhand smoke, and dual users and cigarette only users were classified according to the types of cigarettes used in the past and present. Specifically, current smokers were classified as those who had used cigarettes or e-cigarettes within the past month, and past smokers were classified as those who had used cigarettes or e-cigarettes throughout their lifetime but had not used them within the past month.

### Covariates

The covariates in this study included cumulative smoking exposure (0, 0< and ≤30, 30< and ≤60, or >60 pack-years); exposure to passive smoking (yes, no); sex (male, female); age (40–49, 50–59, 60–69, ≥70 years); education level (lower than college, college or higher); occupation (white, pink, blue collar, none); monthly household income quartile (low, middle-low, middle-high, high); marital status (yes, no); alcohol consumption (2–4 times/week, 2–4 times/month, never or occasionally); body mass index (BMI) (<23, 23 ≤ and < 25, 25 ≤ and < 27.5, 27.5 ≤ and < 30, or ≥30 kg/m^2^); muscle strengthening activity (low or high, with high indicating strength exercises such as dumbbells, weights, and iron bars at least twice a week); aerobic activity (low or high, with high indicating >30 min of aerobic activity ≥5 days per week)^15^; and history of diabetes (no, prediabetes, diabetes), hypertension (no, pre-hypertension, hypertension), asthma (no, yes), stroke (no, yes), and myocardial infarction (no, yes). Prediabetes was defined as having a fasting blood sugar of 100–125 mg/dL or hemoglobin A1c of 5.7–6.4%^17^, and pre-hypertension was defined as having a systolic blood pressure of 120 ≤ and <140 mmHg and diastolic blood pressure of 80≤ and <90 mmHg^18^.

### Statistical analysis

A multiple logistic regression analysis was conducted to examine the relationship between smoking behavior and a high risk of OSA using the STOP-Bang score after adjusting for all covariates. The results are given as adjusted odds ratio (AOR) and 95% confidence interval (CI). A subgroup analysis was also conducted to identify the association between dual cigarette use in the past and present with a high risk of OSA after adjusting for covariates. Finally, stratified analyses were performed through multiple logistic regression to examine the association between smoking behavior and high risk of OSA using the 2-stage STOP-Bang score according to sex, aerobic activity, muscle strengthening activity, and BMI.

All analyses were performed using Statistical Analysis Software version 9.4 (SAS Institute, Cary, NC, USA), and a weighted logistic regression analysis was used to treat the complex and stratified sampling design^15^. The KNHANES provided weights for individual-level analysis to ensure that the survey participants represent the entire population of Korea. Two-sided p<0.05 was considered statistically significant. This study followed the Strengthening the Reporting of Observational Studies in Epidemiology (STROBE) reporting guideline for cross-sectional studies^19^.

## RESULTS

Table 1 presents the general characteristics of the study participants. Among the 7350 participants, 417 (5.7%) had a high risk for OSA according to the 2-stage STOP-Bang score. The number of participants who were dual users (e-cigarettes and cigarettes) was 431 (5.9%), cigarette only users were 2520 (34.3%), and never smokers were 4399 (59.9%). A high risk of OSA was observed in 49 (11.4%) of the 431 dual users and in 290 (11.5%) of the 2520 cigarette only users. Among the 4399 never smokers, 78 (1.8%) had a high risk of OSA, according to the STOP-Bang score.

**Table 1:** General characteristics of study subjects, 2019–2021 Korean National Health and Nutrition Examination Survey

*Characteristics*	*STOP-Bang score for OSA*						p*
	*Total*		*Low/intermediate risk*		*High risk*		
	*n*	*%*	*n*	*%*	*n*	*%*	
Total	7350	100	6933	94.3	417	5.7	
**Smoking behavior**							<0.001
Dual user (e-cig + cig)	431	5.9	382	88.6	49	11.4	
Cigarette only user	2520	34.3	2230	88.5	290	11.5	
Never smoker	4399	59.9	4321	98.2	78	1.8	
**Cumulative smoking exposure** (pack-years)							<0.001
0	4517	61.5	4427	98.0	90	2.0	
0< and ≤30	2169	29.5	1935	89.2	234	10.8	
30< and ≤60	574	7.8	495	86.2	79	13.8	
>60	90	1.2	76	84.4	14	15.6	
**Exposure to secondhand smoke**							<0.001
No	6355	86.5	6030	94.9	325	5.1	
Yes	995	13.5	903	90.8	92	9.2	
**Sex**							<0.001
Male	3226	43.9	2845	88.2	381	11.8	
Female	4124	56.1	4088	99.1	36	0.9	
**Age** (years)							0.001
40–49	1748	23.8	1658	94.9	90	5.1	
50–59	1884	25.6	1754	93.1	130	6.9	
60–69	1980	26.9	1854	93.6	126	6.4	
≥70	1738	23.6	1667	95.9	71	4.1	
**Education level**							0.095
Lower than college	5013	68.2	4744	94.6	269	5.4	
College or higher	2337	31.8	2189	93.7	148	6.3	
**Region**							0.075
Rural	4133	56.2	3881	93.9	252	6.1	
Metropolitan	3217	43.8	3052	94.9	165	5.1	
**Employment**							<0.001
White collar	1474	20.1	1353	91.8	121	8.2	
Pink collar	921	12.5	882	95.8	39	4.2	
Blue collar	1987	27.0	1843	92.8	144	7.2	
None	2968	40.4	2855	96.2	113	3.8	
**Household income**							0.051
Low	1524	20.7	1455	95.5	69	4.5	
Middle low	1819	24.7	1721	94.6	98	5.4	
Middle high	1961	26.7	1830	93.3	131	6.7	
High	2046	27.8	1927	94.2	119	5.8	
**Marital status**							0.001
Not married	1699	23.1	1632	96.1	67	3.9	
Married	5651	76.9	5301	93.8	350	6.2	
**Alcohol consumption**							<0.001
2–4 times/week	1430	19.5	1284	89.8	146	10.2	
2–4 times/month	1341	18.2	1249	93.1	92	6.9	
Never or occasionally	4579	62.3	4400	96.1	179	3.9	
**BMI** (kg/m^2^)							<0.001
<23	2712	36.9	2681	98.9	31	1.1	
23≤ and <25	1801	24.5	1746	96.9	55	3.1	
25≤ and <27.5	1670	22.7	1558	93.3	112	6.7	
27.5≤ and <30	744	10.1	631	84.8	113	15.2	
≥30	423	5.8	317	74.9	106	25.1	
**Aerobic activity**							0.136
Low	5984	81.4	5633	94.1	351	5.9	
High	1366	18.6	1300	95.2	66	4.8	
**Muscle strengthening activity**							0.677
Low	5788	78.7	5463	94.4	325	5.6	
High	1562	21.3	1470	94.1	92	5.9	
**Diabetes**							<0.001
No	2210	30.1	2153	97.4	57	2.6	
Prediabetes^a^	3613	49.2	3414	94.5	199	5.5	
Diabetes	1527	20.8	1366	89.5	161	10.5	
**Hypertension**							<0.001
No	2527	34.4	2493	98.7	34	1.3	
Pre-hypertension^b^	1788	24.3	1726	96.5	62	3.5	
Hypertension	3035	41.3	2714	89.4	321	10.6	
**Asthma**							0.510
No	7109	96.7	6708	94.4	401	5.6	
Yes	241	3.3	225	93.4	16	6.6	
**Stroke**							0.411
No	7150	97.3	6747	94.4	403	5.6	
Yes	200	2.7	186	93.0	14	7.0	
**Myocardial infarction**							0.001
No	7235	98.4	6833	94.4	402	5.6	
Yes	115	1.6	100	87.0	15	13.0	

* Chi-squared test. cig: conventional cigarette. e-cig: electronic cigarette. BMI: body mass index. OSA: obstructive sleep apnea.

a Fasting blood sugar of 100–125 mg/dL or HbA1c of 5.7–6.4%.

b Systolic blood pressure:120≤ and <140 mmHg, and diastolic blood pressure: 80≤ and <90 mmHg.

Table 2 shows the factors associated with a high risk of OSA through multiple logistic regression analysis. Compared to those who were never smokers, cigarette only users and dual users had higher risks of increased OSA (cigarette only users, AOR=2.75; 95% CI: 1.33–5.68 and dual users, AOR=2.45; 95% CI: 1.04–5.79). Other factors that increased the probability of high risk for OSA were male sex, obesity, a history of diabetes, and hypertension.

**Table 2:** Factors associated with high risk of obstructive sleep apnea assessed by STOP-Bang score, 2019–2021 Korean National Health and Nutrition Examination Survey

*Variables*	*High risk of OSA*		
	*AOR*	*95% CI*	p*
**Smoking behavior**			
Dual user (e-cig + cig )	2.45	1.04–5.79	0.042
Cigarette only user	2.75	1.33–5.68	0.006
Never smoker (Ref.)	1.00		
**Cumulative smoking exposure** (pack-years)			
0 (Ref.)	1.00		
0< and ≤30	0.74	0.37–1.51	0.407
30< and ≤60	0.67	0.31–1.44	0.306
>60	1.20	0.44–3.24	0.722
**Exposure to secondhand smoke**			
No	1.00		
Yes	1.30	0.92–1.83	0.141
**Sex**			
Male	9.40	5.61–15.75	<0.001
Female (Ref.)	1.00		
**Age** (years)			
40–49 (Ref.)	1.00		
50–59	1.93	1.32–2.81	0.001
60–69	1.50	0.97–2.32	0.066
≥70	1.03	0.62–1.70	0.980
**Education level**			
Lower than college	1.16	0.84–1.59	0.362
College or higher (Ref.)	1.00		
**Region**			
Rural	1.16	0.88–1.54	0.298
Metropolitan (Ref.)	1.00		
**Employment**			
White collar	1.67	1.08–2.59	0.023
Pink collar	1.01	0.60–1.69	0.978
Blue collar	0.87	0.58–1.30	0.505
None (Ref.)	1.00		
**Household income**			
Low	1.11	0.67–1.81	0.692
Middle low	1.09	0.74–1.61	0.658
Middle high	1.32	0.92–1.89	0.130
High (Ref.)	1.00		
**Marital status**			
Not married	0.80	0.55–1.15	0.220
Married (Ref.)	1.00		
**Alcohol consumption**			
2–4 times/week	1.08	0.74–1.60	0.683
2–4 times/month (Ref.)	1.00		
Never/occasionally	1.31	0.90–1.91	0.159
**BMI** (kg/m^2^)			
<23 (Ref.)	1.00		
23 ≤ and <25	1.15	0.66–2.01	0.624
25≤ and <27.5	3.07	1.88–5.01	<0.001
27.5≤ and <30	6.66	4.10–10.82	<0.001
≥30	17.10	9.83–29.75	<0.001
**Aerobic activity**			
Low	1.11	0.77–1.59	0.590
High (Ref.)	1.00		
**Muscle strengthening activity**			
Low	1.04	0.73–1.47	0.842
High (Ref.)	1.00		
**Diabetes**			
No (Ref.)	1.00		
Prediabetes^a^	0.93	0.64–1.36	0.715
Diabetes	1.73	1.16–2.58	0.007
**Hypertension**			
No (Ref.)	1.00		
Pre-hypertension^b^	1.58	0.94–2.67	0.086
Hypertension	5.57	3.61–8.59	<0.001
**Asthma**			
No (Ref.)	1.00		
Yes	0.98	0.50–1.92	0.963
**Stroke**			
No (Ref.)	1.00		
Yes	0.77	0.37–1.62	0.494
**Myocardial infarction**			
No (Ref.)	1.00		
Yes	1.13	0.59–2.16	0.706

* Multiple logistic regression. AOR: adjusted odds ratio; adjusted for all variables in the table. OSA: obstructive sleep apnea. cig: conventional cigarette. e-cig: electronic cigarette. BMI: body mass index.

a Fasting blood sugar of 100–125 mg/dL or HbA1c of 5.7–6.4%.

b Systolic blood pressure: 120≤ and <140 mmHg, and diastolic blood pressure: 80≤ and <90 mmHg.

**Figure 1:**
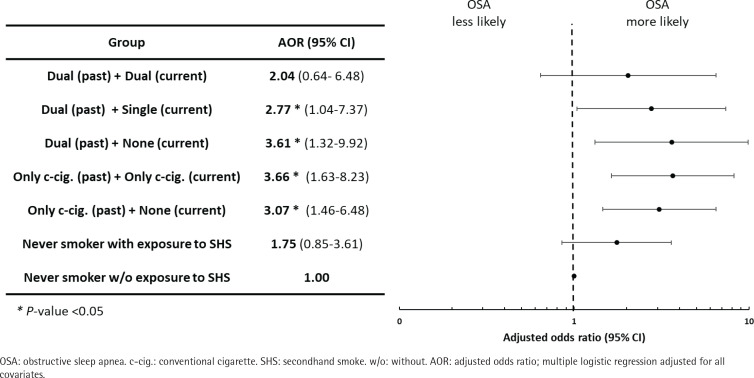
Subgroup analysis of the association of previous and current cigarette type with the risk of obstructive sleep apnea using STOP-Bang score

Figure 1 shows the results of the subgroup analysis. Individuals who did not currently smoke but had a history of dual use were associated with a significant risk of OSA (AOR=3.61; 95% CI: 1.32–9.92). Furthermore, former cigarette smoking was associated with the likelihood of OSA (AOR=3.66; 95% CI: 1.63–8.23). Individuals who were dual users in the past and the present had increased odds of OSA, although this was not statistically significant.

Table 3 presents the results of the stratified analyses according to the independent variables. The probability of a high risk of OSA was 7.35 times higher for females with dual use (AOR=7.35; 95% CI: 1.04–59.80), but this was not significant for males with dual use. The association between the dual use of e-cigarettes and cigarettes and OSA was prominent in the those with low aerobic activity (AOR=2.04; 95% CI: 1.11–3.76) and low muscle strengthening activity (AOR=2.27; 95% CI: 1.17–4.37), and those with high-risk obesity (BMI >30 kg/m^2^) (AOR=4.98; 95% CI: 1.47–16.86). We observed a significant multiplicative interaction between smoking behavior and BMI (p<0.001).

**Table 3:** Stratified analyses of a high risk of OSA versus smoking behavior, according to independent variables, 2019–2021 Korean National Health and Nutrition Examination Survey

*Variables*	*High risk of OSA using STOP-Bang score*						
	*Smoking behavior*						
	*Never smoker (Ref.)*	*Cigarette only user*		p*	*Dual user (e-cig + cig)*		p*
	*AOR*	*AOR*	*95% CI*		*AOR*	*95 % CI*	
**Sex**							
Male	1.00	1.97	1.31–2.97	0.001	1.67	0.96–2.90	0.070
Female	1.00	2.15	0.70–6.58	0.178	7.35	1.04–59.80	0.048
**Aerobic activity**							
Low	1.00	2.07	1.34–3.20	0.001	2.04	1.11–3.76	0.023
High	1.00	2.86	1.00–8.20	0.048	0.66	0.14–3.14	0.598
**Muscle strengthening activity **							
Low	1.00	2.34	1.49–3.68	0.001	2.27	1.17–4.37	0.015
High	1.00	1.20	0.56–2.58	0.647	0.75	0.24–2.38	0.621
**BMI** (kg/m^2^)							
<25	1.00	1.75	0.82–3.72	0.145	0.86	0.27–2.72	0.801
25≤ and <30	1.00	1.90	1.14–3.16	0.014	1.55	0.73–3.28	0.255
≥30	1.00	2.90	1.33–6.31	0.008	4.98	1.47–16.86	0.009

* Multiple logistic regression. AOR: adjusted odds ratio; adjusted for all covariates. OSA: obstructive sleep apnea. cig: conventional cigarette. e-cig: electronic cigarette. BMI: body mass index.

## DISCUSSION

We investigated the association between the STOP-Bang score, which reflects the risk of OSA, and smoking behavior, including smoking status and types of cigarettes (e-cigarettes or cigarettes). The odds of having a high risk for OSA in the dual users and the cigarette only users were significantly higher than in the never smokers. Interestingly, we observed a significantly increased risk for OSA in participants who did not currently smoke but had previously were dual users or cigarette only users.

Several hypotheses may explain our result concerning cigarettes, although the mechanisms are still unclear^13^. First, alterations in sleep architecture caused by smoking may increase the risk of OSA. This hypothesis was based on a prospective study using electroencephalogram spectrum analysis, which found that smokers’ sleep quality was inferior to that of non-smokers, especially in the early stages of sleep^20^. Another study using polysomnography showed that smokers had a higher rapid eye movement sleep density, shorter sleep time, and longer sleep latency^21^. Second, the impairment of upper airway neuromuscular function in smokers can lead to a higher risk of OSA. However, the relationship between nicotine use and upper airway damage has only been demonstrated in animal experiments^22^. In humans, it is unclear whether smoking causes upper airway damage, although it has been shown that smoking can reduce reflexive mechanisms after damage^23^. Third, arousal mechanisms may increase the risk of developing OSA. Smokers have a higher arousal index, which can result in increased sleep instability^24^. Lastly, upper airway inflammation can lead to a higher risk of OSA. Smoking can cause airway inflammation and, consequently, upper airway narrowing^25^.

Although the association between smoking and OSA has been investigated, only a few studies have addressed the association between dual use of e-cigarettes and cigarettes and OSA. However, as more people are using e-cigarettes to quit smoking, and OSA is a growing health problem, awareness of the need for studies on the relationship between e-cigarettes and OSA is gradually increasing. Therefore, in this study, in addition to the association between cigarette smoking and a higher likelihood of OSA, we investigated the association between dual use of cigarettes and OSA. E-cigarettes cause various health problems because e-cigarette aerosols contain highly oxidized freebase nicotine^26^. Additionally, flavoring materials in e-cigarettes are associated with cytotoxicity^27^, and toxic compounds such as carbonyls, metals, volatile organic compounds, and nitrosamines are found in almost all e-cigarettes^28,29^. Nicotine withdrawal can also lead to sleep instability, which can predispose patients to upper airway obstruction^30^. However, considering that our study investigating the association between dual use and the risk of OSA is a cross-sectional study and that the dual users may include many e-cigarette experimenters, the above interpretation requires caution. Cohort studies that can ensure causality are needed.

Despite the knowledge of the effects of e-cigarettes, to the best of our knowledge, Brett et al.^31^ in 2020 were the first to investigate the association between e-cigarettes and sleep disturbance; therefore, the relationship between e-cigarettes and sleep disorders and the mechanism by which e-cigarette use causes sleep disorders is still unclear. According to a previous study, even the sporadic use of e-cigarettes can cause sleep disturbances due to high nicotine exposure. On average, more e-cigarette smokers take sleeping medications than cigarette smokers^31^. Although these hypotheses support the association of dual use with the high risk of OSA, participants who smoked both e-cigarettes and cigarettes, both in the past and present, tended to have a higher risk of OSA than those who never smoked; however, this was not significant. These results could be due to the small number of respondents in these groups, which may show a tendency but is considered insufficient to draw a significant result. Moreover, dual users of cigarettes and e-cigarettes in the past to the present may not have consumed excessive nicotine due to the control on the amount of smoking^32^. Further research needs to be conducted using data from more participants and e-cigarette users.

In the stratified analyses, the association between dual use and the risk of OSA was much more pronounced in females. Although the risk of OSA is lower in females than in males, there is a need to raise awareness as our study shows that dual use was associated with a higher reported likelihood of OSA in females^1^. Furthermore, the association between dual use and OSA was significant in those with low physical activity. Considering that there was no association between aerobic activity and muscle-strengthening activity levels and OSA, the lack of aerobic or muscle-strengthening activity and the dual use of e-cigarettes and cigarettes may exert a synergistic effect on the development of OSA. This suggests that physical activity can offset the negative effects of smoking on OSA^33^. In participants with a BMI > 30 kg/m^2^, the standard of obesity presented by the World Health Organization, mixing the types of tobacco increased the odds of having OSA by approximately five times. The strength of this association was much higher than in those with low BMI, and much higher than in cigarette only users. These results suggest that substances such as cytokines or inflammatory markers released from dual use of cigarettes can affect airway inflammation in obese individuals^34^.

### Limitations

This study has some limitations. First, because a cross-sectional design was used, it was not possible to confirm a causal relationship. Second, the number of e-cigarette users was too small to obtain high statistical power. Third, the number of current dual users was small, making analysis difficult. Fourth, although the cumulative use of cigarettes was included in the analysis, the cumulative use of e-cigarettes was not included because of data limitations. Since e-cigarette experimenters are likely to account for the majority of e-cigarette users, cumulative use of e-cigarettes needs to be calibrated. Fifth, we investigated the association between dual use and the high-risk group for OSA through the STOP-Bang score, not using the diagnosis of OSA directly. Finally, the cutoff for dividing the risk of the STOP-Bang score differs between studies. However, we selected a high-risk group for OSA in 2 steps, and the method was found to have high validity^17^.

## CONCLUSIONS

There are insufficient studies on the association between the types of cigarettes used and OSA, and the association between the two has not been fully elucidated. In the current study, we found that the dual use of e-cigarettes and cigarettes was significantly associated with OSA when compared to non-smoking. In addition, the dual use of cigarettes in the past was associated with a significant likelihood of OSA, even if the participants did not currently smoke or currently smoked only one product type. The association between dual use and OSA was more pronounced in females, participants with obesity, and those with a low level of physical activity. Further research is needed to confirm the causal relationship between the dual use of cigarettes and the risk of OSA.

## Data Availability

The data supporting this research are available from the following source: https://knhanes.kdca.go.kr/knhanes/eng/index.do
